# The Application of a Three-Step Serum Proteome Analysis for the Discovery and Identification of Novel Biomarkers of Hepatocellular Carcinoma

**DOI:** 10.1155/2012/623190

**Published:** 2012-08-16

**Authors:** Asako Kimura, Kazuyuki Sogawa, Mamoru Satoh, Yoshio Kodera, Osamu Yokosuka, Takeshi Tomonaga, Fumio Nomura

**Affiliations:** ^1^Department of Molercular Diagnosis, Graduate School of Medicine, Chiba University, Chiba 260-0856, Japan; ^2^Clinical Proteomics Research Center, Chiba University Hospital, Chiba, Japan; ^3^Laboratory of Biomolecular Dynamics, Department of Physics, Kitasato University School of Science, Kanagawa, Japan; ^4^Department of Medicine and Clinical Oncology, Graduate School of Medicine, Chiba University, Chiba 260-0856, Japan; ^5^Laboratory of Proteome Research, National Institute of Biomedical Innovation, Osaka, Japan

## Abstract

The representative tumor markers for HCC, AFP, and PIVKA-II are not satisfactory in terms of sensitivity and specificity in the early diagnosis of HCC. In search for novel markers for HCC, three-step proteome analyses were carried out in serum samples obtained from 12 patients with HCC and 10 with LC. As a first step, serum samples were subjected to antibody-based immunoaffinity column system that simultaneously removes twelve of abundant serum proteins. The concentrated flow-through was then fractionated using reversed-phase HPLC. Proteins obtained in each fraction were separated by SDS-PAGE. Serum samples obtained from patient with HCC and with LC were analyzed in parallel and their protein expression patterns were compared. A total of 83 protein bands were found to be upregulated in HCC serum. All the protein bands, the intensity of which was different between HCC and LC groups, were identified. Among them, clusterin was most significantly overexpressed (*P* = 0.023). The overexpression of serum clusterin was confirmed by ELISA using another validation set of HCC samples. Furthermore, serum clusterin was elevated in 40% of HCC cases in which both AFP and PIVKA-II were within their cut-off values. These results suggested that clusterin is a potential novel serum marker for HCC.

## 1. Introduction

Hepatocellular carcinoma (HCC) is one of the most common cancers in the world and is a leading cause of death in many countries. Chronic infection by hepatitis B virus (HBV) or hepatitis C virus (HCV) and cirrhosis are major risk factors for HCC development [1, 2]. At present, HCC surveillance with tumor markers and imaging studies such as ultrasonography (US), computed tomography (CT), and magnetic resonance imaging (MRI) have been recommended for patients with cirrhosis [3, 4]. These imaging studies are expensive and the ultrasound is highly dependent on the ability of the operator. Therefore, more sensitive and specific serum biomarkers for early detection of HCC are desirable.

Serum tumor markers for detecting HCC could be divided into 4 categories: oncofetal and glycoprotein antigens, enzymes and isoenzymes, genes, and cytokines. Alpha-fetoprotein (AFP) and protein induced by vitamin-K absence or antagonist-II (PIVKA-II) also called des-gamma-carboxyprothrombin (DCP) are representative tumor markers for the diagnosis of HCC.

The elevated level of AFP is observed in only 50–70% of patients with HCC and also frequently in patients with cirrhosis or exacerbations of chronic hepatitis [5], and its sensitivity is low in patients with earlier/small tumors [6–8]. Measurement of lectin lens culinaris agglutinin (LCA) bound fraction of AFP (AFP-L3) can improve the specificity of AFP. Elevated DCP activity was only present in 28–47.6% of HCCs of less than 3 cm in size [9–11]. Therefore, there has been growing interest and need to develop novel HCC serum biomarkers with greater sensitivity and specificity. Recent studies indicate that some other tumor markers, such as glypican 3 [12–17], gamma-glutamyl transferase II [18], alpha-1-fucosidase [19, 20], vascular endothelial growth factor [21–23], and transforming growth factor-beta 1 [24, 25] could serve as a complementary marker for AFP. Furthermore, the circulating genetic markers such as AFP-mRNA [26, 27] and human telomerase reverse transcriptase mRNA [28, 29] have been shown to be diagnostic and prognostic indicators of HCC.

Proteomics is the systematic study of proteomes, which describes the complete set or proteins found in a given cell type as well as of body fluids such as serum and urine. Recent advances in sophisticated technologies in proteomics should provide promising ways to discover novel markers in various fields of clinical medicine.

Increasing number of recent reports provide evidence that proteomic approach is promising tools to discover and identify novel biomarker for HCC. In particular, surface-enhanced laser desorption/ionization time-of-flight mass spectrometry (SELDI-TOF MS) is a representative example of a proteomics technique for the high-throughput fingerprinting of serum proteins and peptides [30]. We used the SELDI technology to generate comparative protein profiles of consecutive serum samples obtained during abstinence from alcoholic patients and found some novel biomarker for excessive alcohol consumption [31, 32]. Using this technique, several protein peaks leading to differentiation of patients with HCC from patients with cirrhosis alone have been discovered [33, 34]. In these studies, crude serum samples were directly analyzed without particular preanalytical preparations. The technical challenge in the analysis of serum proteome is that serum proteins are present at unequal concentrations. Indeed, 22 of the most abundant proteins account for >99% of total serum proteins [35], which hampers the detection of thousands of other low abundance proteins and peptides.

To detect the disease-associated proteins present in low abundance using currently available methods, the most abundant proteins have to be removed first by technique such as immunodepletion. We recently developed a three-step serum proteome analysis involving removal of 12 abundant proteins and subsequent reversed-phase high-performance liquid chromatography fractionation and one-dimensional electrophoresis and identified three proteins including YKL-50 as a promising biomarker of sepsis [36]. More recently, using this method, we identified promising biomarkers for alcohol abuse [37], breast cancer [38], and pancreatic cancer [39].

 In this study, we applied this three-step proteome analysis to find novel biomarkers of HCC.

## 2. Material and Methods

### 2.1. Patients and Serum Samples

As an initial set of samples, blood samples of 12 HCV-related HCC patients and 10 HCV-related LC patients obtained at the Department of Medicine and Clinical Oncology, Chiba University Hospital, were used for comprehensive proteome analysis. All patients were positive for hepatitis C antibodies on the day of sampling and were diagnosed pathologically or clinically. 

Diagnostic values of marker candidates identified in the initial set of samples were further validated using another set of samples. For this purpose, serum sample sets of 64 HCV-related HCC and 60 HCV-related LC patients were obtained from Chiba University Hospital and 60 healthy individuals for normal control from Kashiwado Clinic in Port-Square of Kashiwado Memorial Foundation, Chiba. These healthy individuals were defined in this study as subjects without medication on a regular basis, obesity, heavy drinking, abnormal liver test results, and hepatitis virus carriage.

Written informed consent was obtained from all patients. Serum samples were obtained and processed under the standardized conditions as we reported elsewhere [40] and were stored as aliquots at −80°C until analysis.

The clinical characteristics of all the patients are shown in Tables 1 and 2.

### 2.2. Immunoaffinity Depletion of High-Abundant Proteins from Human Serum

**Figure 1:**
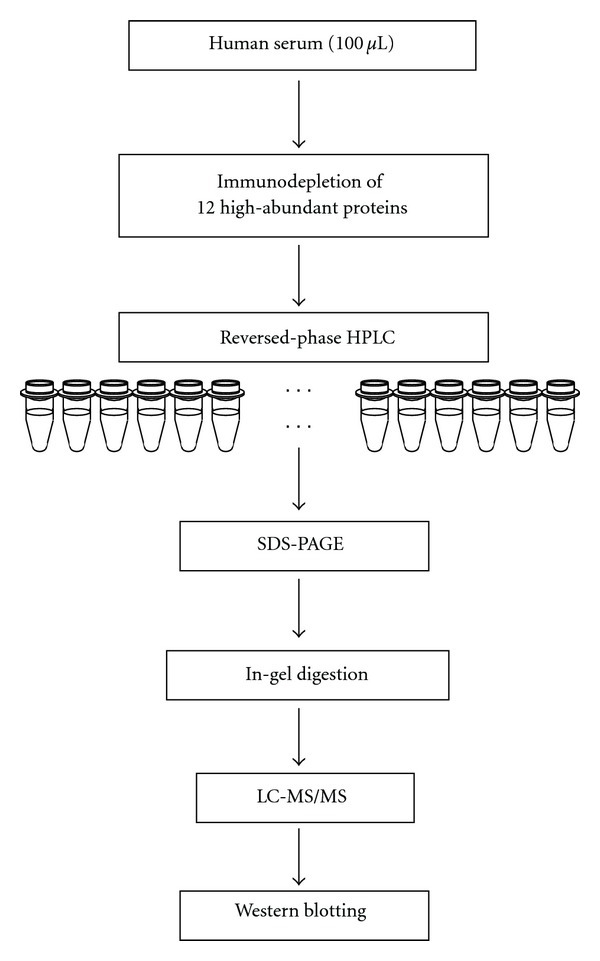
Schematic diagram of the experimental protocol.

The outline of our experimental procedures is summarized in Figure 1. For removal of the twelve most abundant proteins: albumin, IgG, transferrin, fibrinogen, IgA, aplha2-macroglobrin, IgM, aplha1-antitrypsin, haptoglobin, aplha1-acidglycoprotein, apolipoprotein A-I, and apolipoprotein A-II, Proteome Lab IgY-12HC LC10 column (Beckman coulter Inc., Fullerton, CA, USA) was used. According to the manufacturer's instructions, 100 *μ*L of each serum was diluted 5-fold with buffer A (dilution buffer) and injected onto the column in 100% buffer A at a flow rate of 0.5 mL/min for 25.0 min and 2.0 mL/min for 5.0 min on a Shimadzu LC10A VP system (Shimadzu Co., Kyoto, Japan). After collection of the flow-through fraction containing unbound proteins, the column was washed and the bound proteins were eluted with 100% buffer B (stripping buffer) at a flow rate of 2.0 mL/min for 18.0 min.

The chromatograms were monitored at 280 nm and 8 fractions (flow-through) were collected at 0.5 min intervals from 12.1 to 20.0 min. The fractions were collected into 1.5 mL microcentrifuge tubes.

### 2.3. Concentrating of Fractions by Centrifugal Ultrafiltration

The flow-through fractions (total 4.0 mL) were applied to Vivaspin 2 spin concentrators (MWCO 10 KD, Vivascience, Hannover, Germany) and concentrated to a volume of 80 *μ*L according to the manufacturer's instructions. The concentrated pool was stored at −80°C until use.

### 2.4. HPLC Sample Preparation, Separation, and Fraction Collection

HPLC separations were performed on an automated SHISEIDO NANOSPACE SI-2 system (Shiseido Fine Chemicals, Tokyo, Japan). Injection was performed by an autosampler with a completely filled 100 *μ*L injection loop. 75 *μ*L of concentrated flow-through samples were directly loaded onto the Intrada WP-RP column (Imtakt, Kyoto, Japan). The RP separations for each flow-through were performed under a set of conditions using a multisegment elution gradient, with eluent A (0.1% TFA in water, v/v) and eluent B (0.08% TFA in 90% acetonitrile, v/v). The gradient conditions consisted of three steps with increasing concentrations of the eluent B: 5% B 5 min, 5–95% B 23 min, 95% B 11 min, and 5% B 21 min for reequilibration of the column, at a flow rate of 0.40 mL/min for a total runtime of 60 min.

The chromatograms were monitored at 218 nm and 40 fractions were collected at 0.5 min intervals from 19.1 to 39.1 min. Each fraction was dried in a centrifugal vacuum concentrator and stored at −80°C for subsequent SDS-PAGE analysis.

### 2.5. Electrophoretic Analysis

Dried fraction samples from HPLC separations were dissolved in 15 *μ*L of sample preparation buffer, vortexed, and then loaded onto the two Perfect NT Gels (10–20% acrylamide, 20 wells, 140 mm × 140 mm × 1 mm; DRC. Co., Ltd.).

SDS-PAGE analysis was carried out by an established method [41]. Following electrophoresis, proteins were visualized by silver staining using 2D silver stain II “DAIICHI” (Daiichi Pure Chemicals Co., Ltd., Osaka, Japan).

### 2.6. In-Gel Digestion

For protein identification, samples were prepared again as described above. To obtain high sensitivity, the same process was repeated three times per sample; finally dried fraction sample of triple amount were obtained. 45 *μ*L of combined dried fraction samples were loaded on to SDS-PAGE gel as described above after these samples were individually dissolved with 15 *μ*L sample buffer.

After then, protein spots in Coomassie brilliant blue (CBB) stained SDS-PAGE gels were individually excised in squares of about 1 to 2 mm per side destained in 50% v/v acetonitrile/50 mM NH_4_HCO_3_ and then washed with deionized water. The gel pieces were dehydrated in 100% acetonitrile for about 15 min and then dried in a SpeedVac Evaporator (Wakenyaku, Kyoto, Japan) for 60 min. The pieces were rehydrated in 10–20 *μ*L of 25 mM Tris-Cl (pH 9.0) containing 25 ng/*μ*L trypsin (Trypsin sequence grade, Roche, Mannheim, Germany) for 45 min at 4°C. After removal of excess trypsin, the gel pieces were incubated in a minimal volume (10–20 *μ*L) of 50 mM Tris (pH 9.0) buffer for 24 h at 37°C. The solution containing digested fragments of proteins was transferred to 1.5 mL siliconized plastic test tube and stored at 4°C. Peptide fragments remaining in gel pieces were further recovered after 20 min incubations at room temperature in minimal volumes of 5% v/v formic acid containing 50% v/v acetonitrile. The solutions containing peptides were pooled together in the tube at 4°C. 

### 2.7. LC-MS/MS

Molar quantities of recovered peptide fragments were estimated from the staining intensity of the SDS-PAGE bands that were digested in-gel with trypsin. Digested peptides equivalent to the maximum of 10 pmoL of a protein in an SDS-PAGE band were injected into a Magic C18 column (Michrom Bioresources, Inc., CA, USA), which was attached to the MAGIC 2002 (Michrom Bioresources, Inc., CA, USA) high-performance liquid chromatography (HPLC) system. The flow rate of the mobile phase was 1 *μ*L/min using MAGIC Variable Splitter. The solvent composition of the mobile phase was programmed to change in 50 min cycles with varying mixing ratios of solvent A (2% v/v CH_3_CN and 0.1% v/v HCOOH) to solvent B (90% v/v CH_3_CN and 0.1% v/v HCOOH). Next, the peptides were eluted with a linear gradient from 0 to 50% solvent B. Purified peptides were introduced from HPLC to Q-star (Applied Biosystems, Foster City, CA, USA), a hybrid quadrupole time-of-flight mass spectrometer, via an attached FortisTip (AMR, Tokyo, Japan). Mascot search engine (Matrixscience, London, UK) was used to identify proteins from the mass and tandem mass spectra of peptides. Peptide mass data were matched by searching the National Center for Biotechnology Information database using MASCOT engine (http://www.matrixscience.com/). The minimum criterion of the probability-based MASCOT/MOWSE score was set with 5% as the significant threshold level.

### 2.8. Western Blot Analysis

After the 12 abundant proteins were removed from serum as described above, the depleted samples were separated on SDS-polyacrylamide gel electrophoresis (80 × 40 × 1.0 mm, 10–20% polyacrylamide gradient gel, 240 V) and transferred to a methanol-rinsed polyvinyl-difluoride (PVDF) membrane (0.45 *μ*m pore size in roll form, Millipore, Bedford, MA) (Amersham, Hybond-C Extra Supported) (40 V, 25 min) using the XV Pantera System (DRC Co., Ltd., Tama, Japan). After transferring the proteins to a membrane and blocking with 5% skim milk in phosphate-buffered saline (PBS) for 1 h at room temperature, the membranes were incubated at 4°C overnight with the primary antibody to clusterin (1 : 3000, mouse monoclonal, upstate (now part of Millipore), CA, USA). The membrane was washed for a total 30 min in 3 changes of PBS-Tween (0.1%) prior to incubation in the appropriate horseradish peroxidase-linked secondary antibody (anti-mouse IgG horseradish peroxidase-linked secondary antiserum, 1 : 500) for 1 h at room temperature. The membranes were finally washed three times as previously described, and immunoreactive proteins were revealed with an enhanced chemiluminescence substrate reaction using ECL western blotting detection reagents (GE Healthcare UK Ltd., Amersham, England) according to the manufacturer's instructions.

### 2.9. Gel Imaging and Analysis

The Silver-stained SDS-gels and CBB-stained gels were scanned with an optical resolution of 400 dpi by EPSON ES-2000 scanner (SEIKO EPSON Corp., Nagano, Japan) using EPSON TWAIN Pro software (SEIKO EPSON Corp., Nagano, Japan). The images were processed using Photoshop 6 (Adobe) software. After scanning, each gel was stored at 4°C.

TIFF files of the gel images were transferred for analysis with a TotalLab TL120 (Nonlinear Dynamics Ltd., Newcastle, UK) and were used for band detection and statistical analysis.

### 2.10. Measurement of Serum Clusterin Concentration by ELISA. 

Serum clusterin was quantified using a human clusterin ELISA kit (R&D systems, Inc., MN, USA) following manufacturer's instructions. Human clusterin standard as provided in the kit (1,000 ng/mL: stock solution), and the serially diluted standards (200–3.12 ng/mL) were prepared from the stock solution. Calibrator Diluent RD5T (dilution buffer) serves as the blank. Test serum samples were diluted 1 : 2000 in the dilution buffer.

After adding 100 *μ*L of Assay Diluent RD1-19 to each well, 50 *μ*L aliquots of the standards and diluted test samples were added in duplicate to the wells of a microtiter plate coated with antihuman clusterin antibody. 

After incubation at room temperature for 2 hours on a horizontal orbital shaker, the plate was washed using 400 *μ*L of Wash Buffer and repeated three time processes and a total of four washes. After the last wash, 200 *μ*L of antihuman clusterin monoclonal antibody conjugated to horseradish peroxidase was added to the wells. The plate was incubated for 2 hours at room temperature on the shaker, followed by washes as before and addition of 200 *μ*L of substrate solution containing hydrogen peroxide and tetramethylbenzidine to the wells. The plate was stetted at the dark to protect from light and incubated for 30 min at room temperature to allow for color development. The reaction was stopped by the addition of 50 *μ*L of stop solution, and the optical densities were determined by reading absorbance at 450 nm with iMark Microplate Reader (Bio-Rad Laboratories, Inc., CA, USA).

### 2.11. Other Procedures

Numerical data were presented as mean ± SD. Statistical significance of difference was assessed by Student's *t*-test; *P* values less than 0.05 were considered significant.

Serum AFP and PIVKA-II levels were determined by commercially available assay kits.

## 3. Results

### 3.1. Immunoaffinity Serum Depletion

Schematic diagram of our experimental protocol is summarized in Figure 1.

**Figure 2:**
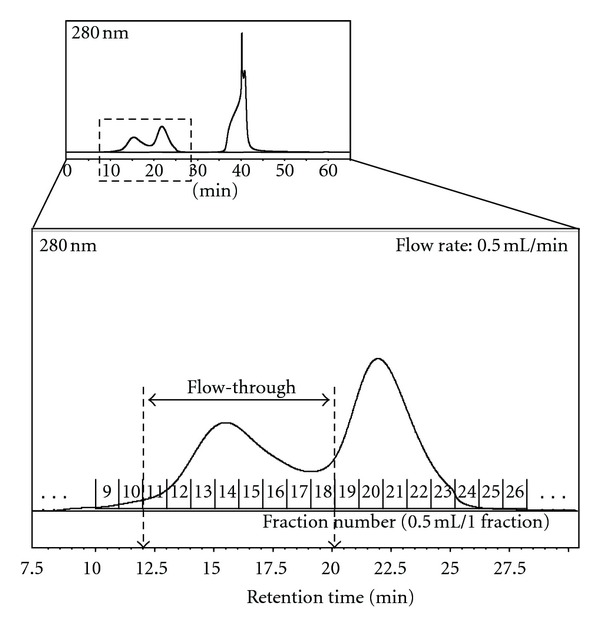
Representative chromatogram during removal of highly abundant serum proteins by an immunoaffinity column. 100 *μ*L of serum (diluted fivefold) was injected on the immunoaffinity column and was eluted (0.5 mL/min) as described in the experimental procedures. Flow-through fractions (12.1–20.0 min) were collected, and then a total of 4.0 mL fractions were concentrated to a volume of 80 *μ*L using Vivaspin 2 for reversed-phase HPLC fractionation.

Figure 2 is a representative immunoaffinity chromatogram and shows a substantial removal of high-abundant proteins from a human serum sample. The immunodepletion of the high-abundant serum proteins was conducted in a reproducible manner in samples obtained from seven HCC and five LC patients (data not shown). A total of 4 mL of flow-through fractions were collected, desalted, and concentrated prior to reversed-phase HPLC.

### 3.2. RP-HPLC

**Figure 3:**
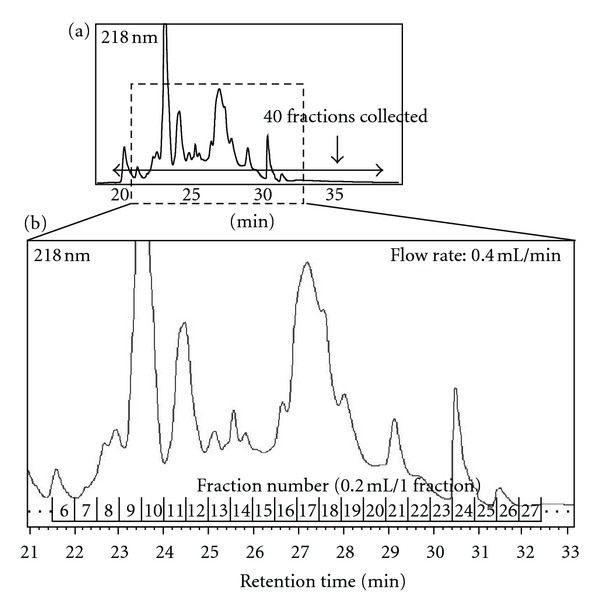
Representative chromatogram during fractionation by RP-HPLC of serum samples in which highly abundant proteins were immunodepleted. Concentrated immunodepleted samples were directly loaded onto the RP column and 40 fractions were collected every 0.5 min from 19.1 to 39.1 min ((a) arrow) as described in the experimental procedures. Among them, a total of 22 fractions were processed for SDS-PAGE analysis (b).

Figure 3 is a representative reversed-phase HPLC chromatogram. Forty fractions were collected every 0.5 minute from 19.1 to 39.1 minutes (Figure 3(a), arrow). Fractions numbers 1–5, numbers 6–8, numbers 26–30, numbers 31–35, and numbers 36–40 were pooled, respectively, since protein concentration of each fraction was apparently very low. Therefore, a total of 22 fractions were processed for SDS-PAGE analysis (Figure 3(b)).

### 3.3. SDS-PAGE Analysis

**Figure 4:**
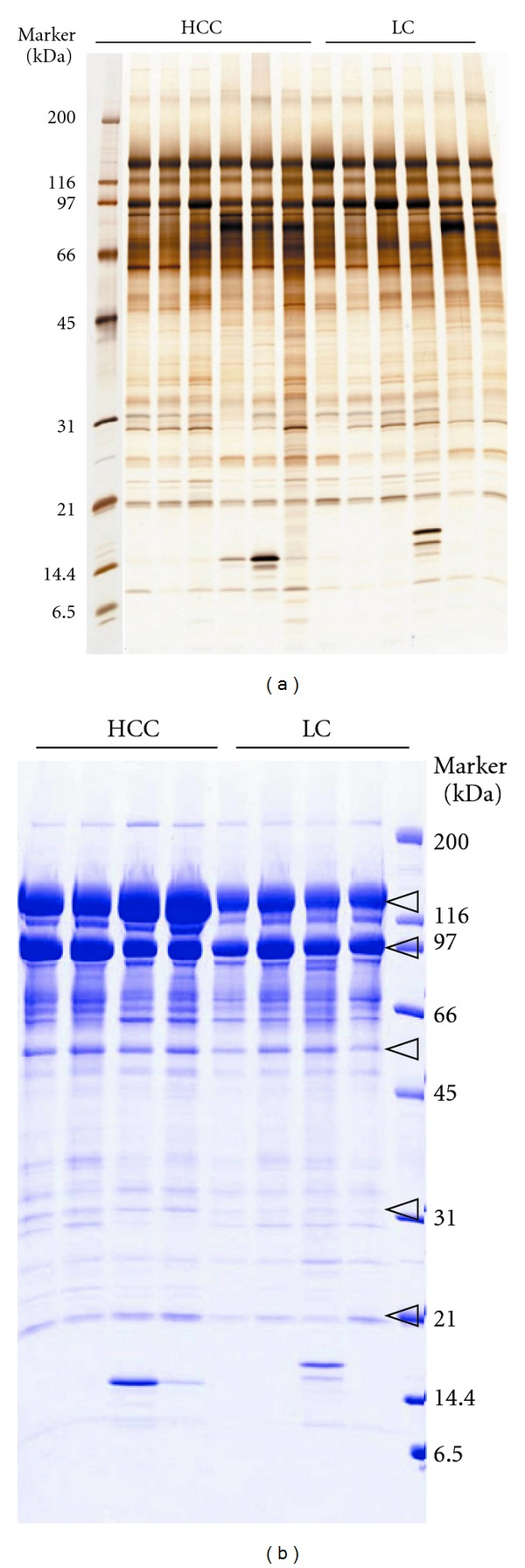
Representative SDS-PAGE pattern of immunodepleted serum sample after RP-HPLC fractionation (fraction number 13). 100 *μ*L of serum samples from seven HCC patients and five LC patients was immunodepleted and injected onto the column. Forty fractions were collected and dried, and among them 22 fractions were separated using 10–20% SDS-PAGE. Each dried fraction was dissolved in 15 *μ*L of sample buffer and loaded onto the gel as described in the experimental procedures. Following electrophoresis, proteins were visualized by silver staining (a). For protein identification, 300 *μ*L of serum samples was prepared again and visualized by CBB staining (b). The intensities of 14 bands were increased in all the seven HCC patients. The representative examples are indicated by arrow heads.

The representative silver-stained SDS-PAGE gel of a fraction (fraction number 13) obtained from seven HCC patients and five LC patients is shown in Figure 4(a).

Comparison of SDS-PAGE patterns of a total of 22 fractions revealed that intensities of 83 bands were greater in more than 3 cases of HCC than in those in LC cases. Among these, the intensities of 14 bands were increased in all the seven HCC patients. The representative examples are indicated by arrow heads.

### 3.4. Identification of Protein

**Figure 5:**
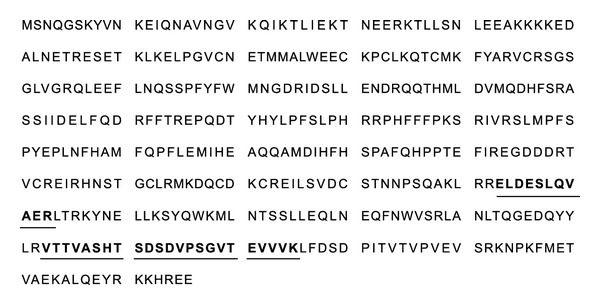
Identification of clusterin by LC-MS/MS. The amino acid sequence of clusterin is shown. Matched peptide sequences are printed in bold and underlined.

To identify proteins, the expression of which was different between HCC and LC on silver stained gel, four HCC and four LC sera were fractionated and separated using SDS-PAGE again, and then gels were stained by CBB (Figure 4(b)). Because the sensitivity of the CBB stain is lower than of the silver stain, samples for identification were prepared from the beginning by repeating three courses of the procedures, from depletion of the major proteins to RP-HPLC fractionation. As a result, additional 71 bands were found to have altered intensity levels between the two groups on CBB gels. Thus, a total of 154 bands were considered as initial candidate bands. Forty-six out of these 154 bands, derived from more than two adjacent fractions, were not processed further. Finally, 108 bands were subjected to in-gel trypsin digestion: among them 73 proteins were identified by LC-MS/MS (Table 3 and Figure 5).

### 3.5. Western Blotting

**Figure 6:**
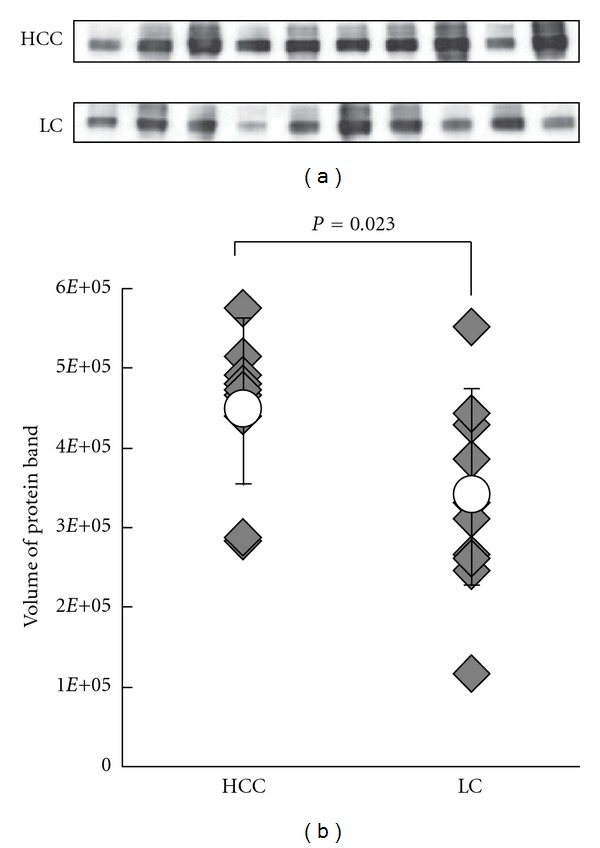
Western blot analysis of clusterin in sera from HCC and LC groups. (a) Immunoedpleted sera of the 5 HCC and 5 LC SDS-PAGE cases and additional 5 HCC and 5 LC cases were separated by 10.0–12.0% SDS-PAGE and probed with anticlusterin. The expression levels are relatively higher in the HCC groups than LCs. (b) Differences in expression were analyzed by the Student's *t*-test. The expression levels of these proteins were upregulated significantly in HCC samples (*P* = 0.023). Rhombuses represent volumes of individual samples. Line indicates the range with the open circles indicating the mean values.

Western blotting analysis could confirm that clusterin was overexpressed in the majority of HCC sera as compared with LC (Figure 6(a)).

Semiquantitative analysis of the results by TotaLab TL120 (Shimadzu Co., Ltd., Kyoto) revealed that the difference in serum clusterin levels between HCC and LC was statistically significant (468211.38 ± 103972.69 versus 341686.90 ± 123162.85, *P* = 0.023) as indicated in Figure 6(b). 

### 3.6. Clusterin Concentration in Serum from HCC and LC Patients

**Figure 7:**
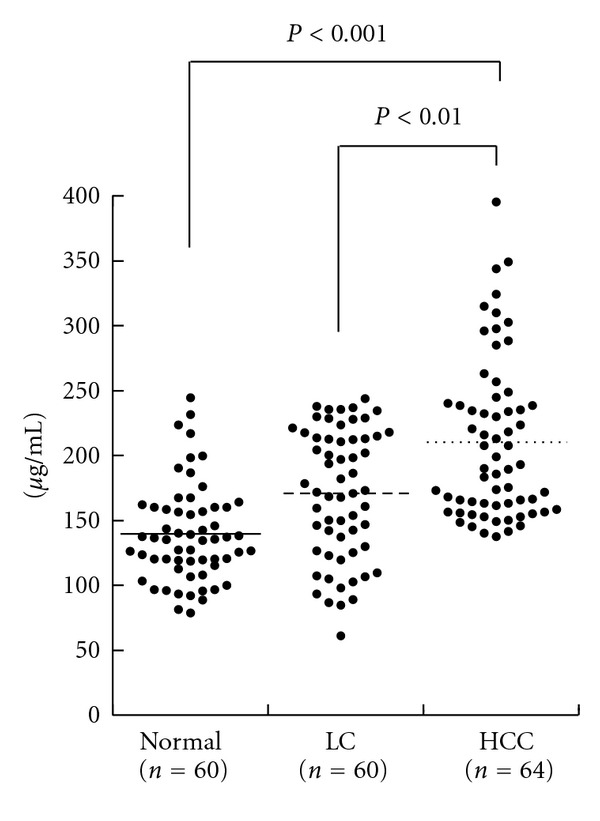
Concentration of clusterin in 64 patients with HCC, 60 patients with LC and 60 healthy individuals. Clusterin levels, quantified using ELISA, were significantly greater in HCC patients compared with LC, (*P* < 0.01) and normal subjects (*P* < 0.001).

To evaluate diagnostic values of serum clusterin levels for HCC diagnosis, we examined sera from 64 patients with HCC, 60 with LC, and 60 normal subjects. The concentration of clusterin (mean ± SD) was 210.4 ± 61.3 *μ*g/mL for HCC, 170.9 ± 50.0 *μ*g/mL for LC, and 139.4 ± 37.4 *μ*g/mL for normal subjects and was significantly higher in HCC than in LC (*P* < 0.01, Student's *t*-test) and in normal subjects (*P* < 0.001) (Figure 7).

We set the cut-off value of clusterin at 230 *μ*g/mL by calculating the mean + 2 SD of healthy 60 samples. As a result, clusterin level above the value was found in 23 of 64 HCCs (35.9%) and in 6 of 60 LCs (10.0%). Furthermore, serum clusterin levels were above the cut-off value in 5 of 12 HCCs (41.7%) in whom both serum AFP and PIVKA-II were within their cut-off values, suggesting that clusterin is complementary to the conventional two representative HCC tumor markers.

## 4. Discussion

The sequencing of the human genome has opened the door for comprehensive transcriptome and proteome analysis. Transcriptome analyses have revealed unique patterns for gene expression that are clinically informative. Messenger RNA abundances, however, are not necessarily predictive of corresponding protein abundances [42]. Since the detailed understanding of biological processes, both in healthy and pathological states, requires the direct study of relevant proteins, proteomics bridges the gap between the information coded in the genome sequence and cellular behavior. Therefore, proteomics is among the most promising technologies for the development of novel diagnostic tools. 

Increasing number of studies has taken advantage of various proteomic technologies to discover and identify novel HCC markers. Clinical tissue samples have been the most extensively studied samples in HCC proteomic studies. Most studies compared protein expression profiles between tumor tissues and adjacent nontumor tissues using two dimensional electrophoresis (2DE) and two dimensional fluorescence difference gel electrophoresis (2D-DIGE). 

Some studies used laser capture microdissection (LCM) in order to characterize isolated tumor cell populations from heterogeneous tissue sections. By combing LCM and 2D-DIGE, Liang et al [43]. found that the protein profiles of well- and poorly differentiated HCC tissues are significantly different. Proteome analyses of tumor tissues should be a basis for HCC marker discovery and a number of proteins have been identified as candidate markers for HCC [44–46]; none of them have been shown to be useful serum marker in a clinical setting. Among thousands of serum proteins and peptides, a few are so dominant that they may hamper the detection of other low abundance proteins or peptides. To overcome this problem, Feng et al. [47] took a strategy to deplete abundant proteins such as albumin and immunoglobulin before analyses, followed by 2DE and MALDI-TOF MS/MS identification. They showed that heat-shock-protein 27 could aid in the diagnosis of HCC.

In this study, three-step procedures including the immunodepletion of 12 abundant proteins were carried out to discover novel HCC markers. As a first step, serum samples were subjected to antibody-based immunoaffinity column that simultaneously removes 12 abundant serum proteins. The concentrated flow-through was then fractionated using reversed-phase HPLC. Proteins obtained in each HPLC fraction were further separated by SDS-PAGE. A total of 73 differentially expressed proteins were identified and among them clusterin was of particular interest as potential serum marker for HCC and differences in this expression in serum were confirmed by the western blotting. 

Further validation using another set of serum sample set showed that clusterin level was significantly higher in HCC than in LC as determined by ELISA. It is notable that serum clusterin levels were elevated in 5 out of 12 HCC cases in which both AFP and PIVKA-II were within their cut-off values. As a result, combination assays of AFP PIVKA-II and clusterin could detect about 90% of HCC cases included in this study. These result suggested that clusterin could be HCC tumor marker complemenatary to AFP and PIVKA-II.

Clusterin, also known as apolipoprotein J (Apo J), sulfated glycoprotein 2, is a heterodimeric glycoprotein present in most animal tissues and body fluids [48]. This glycoprotein plays important roles in a variety of physiological processes including lipid transport [49], reproduction [50], tissue remodeling [51], and senescence [52].

Clusterin overexpression has been shown in various human malignancies including cancer of the breast [53], pancreas [54], and colon [55]. Kang et al. [56] demonstrated the overexpression of clusterin in HCC and suggested that its cytoplasmic overexpression might be a predictor of poor survival. Increased serum levels of clusterin in HCC patients had not been reported before.

In conclusion, the results of this study suggest that clusterin can be a supplementary serum biomarker for HCC. Exact mechanisms and pathophysiological significance for the upregulation of clusterin in HCC remain to be investigated. Furthermore, since the majority of HCC cases in Japan are related to HCV, we focused on HCV-related HCC in the present study. It will be necessary to assess diagnostic values of serum clusterin levels in HBV-related cases as well.

## Figures and Tables

**Table:** (a)

Case number	Sex	Age	Stage	Child-Pugh	Tumor size (mm)	Differentiation	AFP (ng/mL)	PIVKA-II (mAU/mL)
H1	M	71	III	A	Multiple, max 30	Poorly	70.6	18
H2	M	73	IV	B	Multiple, max 50	Moderately	549.6	44
H3	F	69	III	A	38	Moderately–well	11.9	59
H4	M	73	III	B	32–30	Moderately	208.1	20
H5	M	69	III	B	29–25	Moderately–well	38.3	12
H6	F	77	III	A	Multiple, max30	Moderately–well	47.3	10
H7	M	67	III	A	30–10	Moderately	14.9	35
H8	F	71	III	B	35–20	Well	1031.7	466
H9	M	58	IV	B	20	Moderately	25.1	75
H10	F	71	III	B	30	Poorly–moderately	14640	<10
H11	M	81	III	A	25	Moderately	62.3	6854
H12	M	70	III	A	60	—	1390	5350

**Table:** (b)

Case number	Sex	Age	Child-Pugh	AFP (ng/mL)	PIVKA-II (mAU/mL)
L1	M	45	B	12	—
L2	F	54	A	36.4	<10
L3	M	59	A	24.3	31
L4	M	59	B	12.8	66
L5	F	62	A	4.5	—
L6	M	43	A	8.5	—
L7	M	48	B	11.3	—
L8	M	60	A	37.7	—
L9	F	68	A	15.3	—
L10	M	45	B	6	—

**Table 2:** Clinical characteristics of 64 patients with HCC, 60 with LC and 60 normal control subjects.

	HCC	LC	Normal control
	*n* = 64	*n* = 60	*n* = 60
Age, mean ± SD	66.1 ± 9.9	56.8 ± 12.3	54.5 ± 7.0
Male/female	7.0	6.5	6.5
AFP level (ng/mL), mean ± SD	1926.2 ± 11904.7	14.7 ± 24.2	3.5 ± 1.6
PIVKA-II level (mAu/mL), mean ± SD	11757.2 ± 50071.5	18.1 ± 12.5	19.71 ± 5.4

**Table 3:** Upregulated proteins identified by LC-MS/MS in human HCC serum. Details are described in the experimental procedures.

Protein name	Theoretical mass	Experimental mass	Score	Coverage	Queries matched
Afamin precursor	69024	90000	563	23%	16
Alpha-1-antichymotrypsin	48606	95000	118	4%	2
Alpha-1-B-glycoprotein	51908	95000	88	5%	2
Alpha-1-microglobulin	16531	230000	99	15%	2
Alpha-2-macroglobulin	163175	170000	2465	34%	76
Angiotensinogen	53122	55000	705	29%	23
Antithrombin III	49008	62000	389	24%	9
Apolipoprotein A-IV	45307	43000	639	30%	12
Apolipoprotein B	187126	140000	332	4%	7
Apolipoprotein B-100	515077	140000	267	1%	5
Apolipoprotein E	36185	35000	832	44%	33
Beta-actin	41710	41000	226	17%	4
C1q	26442	33000	113	11%	2
C2 precursor	83214	97000	557	15%	14
C3	187046	180000	613	7%	11
C3b	103886	120000	2042	26%	63
C3c	187046	33000	206	2%	4
C3d	120000	187046	153	1%	3
C3, isoform CRA_a	70000	143619	1636	28%	64
C4A	192741	24000	456	5%	21
C4B	40737	41000	138	7%	2
C5	141723	120000	248	4%	6
C6	104776	120000	96	4%	2
C7	93449	95000	874	22%	27
C8-alpha subunit	65111	55000	109	3%	2
C8-beta propeptide	62008	70000	323	11%	7
C8-gamma polypeptide	22206	23000	352	40%	15
C9	60359	70000	496	14%	12
Complement receptor type 2 (Cr2)	112900	160000	160	2%	2
Component factor B	85450	50000	163	4%	3
Cr2-C3d complex	34437	35000	151	10%	3
Complement component (3b/4b) receptor 1	61415	160000	102	4%	2
Carbonic anhydrase I	28852	25000	195	18%	4
Carbonic anhydrase II	29200	33000	208	18%	4
Carboxypeptidase N Polypeptide 1 precursor	52253	45000	147	11%	3
Carboxypeptidase N precursor	60578	97000	330	11%	6
Cathepsin D preproprotein	44524	49000	103	6%	2
Cationic trypsinogen	160000	120901	66	—	2
CD14 protein precursor	40111	55000	365	21%	8
Ceruloplasmin	115398	140000	1520	27%	44
Clusterin	48772	39000	95	8%	2
C-type lectin domain family 3, member B	22552	25000	243	24%	6
Fibronectin precursor	256529	230000	176	2%	3
Galectin 3 binding protein	65289	90000	177	3%	3
Gelsolin	85644	95000	474	15%	11
Glutathione peroxidase 3 precursor	25489	26000	167	16%	4
Hemopexin precursor	51512	50000	104	4%	2
Heparin cofactor II	57034	110000	372	21%	8
Insulin-like growth factor binding protein	65994	95000	176	6%	3
Interalpha-trypsin inhibitor heavy chain H1	101339	230000	631	14%	23
Interalpha-trypsin inhibitor heavy chain H2	106370	230000	862	15%	28
ITI family heavy chain-related protein	103321	120000	348	9%	6
Kininogen 1	47853	120000	355	13%	7
Lactate dehydrogenase B	36615	35000	217	13%	4
Leucine-rich alpha-2-glycoprotein 1	38154	50000	301	15%	8
Lumican	38375	70000	320	21%	7
M130 antigen	160000	120901	66	1%	2
Multimerin 2	104352	20000	88	1%	2
Pancreatic carboxypeptidase A1 precursor	47111	41000	92	5%	2
Peptidoglycan recognition protein 2 precursor	67927	70000	96	7%	2
Pigment epithelial-differentiating factor (serpin-F1)	46313	48000	946	50%	22
Plasma protease (C1) inhibitor precursor	55147	90000	829	25%	43
Prepro-plasma carboxypeptidase B	48411	62000	100	6%	2
Preserum amyloid P component	25381	30000	362	26%	13
Prolidase	54348	55000	82	3%	2
Proteasome alpha 4 subunit isoform 1	29465	33000	85	8%	2
Sex-hormone-binding globulin	43768	45000	391	28%	10
Thyroxine-binding globulin precursor	46295	55000	135	6%	3
Trypsin inhibitor	106647	55000	174	5%	3
Vascular cell adhesion molecule 1 isoform	81224	97000	133	3%	2
Vitamin D binding protein	51183	55000	669	21%	17
Vitamin K-dependent protein S	75074	95000	106	3%	2
Vitronectin	54308	10000	90	2%	2

## List of Abbreviations

HCC: Hepatocellular carcinoma
AFP: Alpha-fetoprotein
DCP: Des-gamma-carboxy prothrombin
LC: Liver cirrhosis
SDS-PAGE: Sodium dodecyl sulfate polyacrylamide gel electrophoresis.
